# Connexin 43 impacts on mitochondrial potassium uptake

**DOI:** 10.3389/fphar.2013.00073

**Published:** 2013-06-06

**Authors:** Kerstin Boengler, Elvira Ungefug, Gerd Heusch, Luc Leybaert, Rainer Schulz

**Affiliations:** ^1^Physiologisches Institut, Justus-Liebig-Universität GiessenGiessen, Germany; ^2^Institut für Pathophysiologie, Universitätsklinikum EssenEssen, Germany; ^3^Department of Basic Medical Sciences, Ghent UniversityGhent, Belgium

**Keywords:** connexin 43, mitochondria, potassium uptake, Gap19, PBFI

## Abstract

In cardiomyocytes, connexin 43 (Cx43) forms gap junctions and unopposed hemichannels at the plasma membrane, but the protein is also present at the inner membrane of subsarcolemmal mitochondria (SSM). Both inhibition and genetic ablation of Cx43 reduce ADP-stimulated complex 1 respiration. Since mitochondrial potassium influx impacts on oxygen consumption, we investigated whether or not inhibition or ablation of mitochondrial Cx43 alters mitochondrial potassium uptake. SSM were isolated from rat left ventricular myocardium and loaded with the potassium-sensitive dye PBFI (potassium-binding benzofuran isophthalate). Intramitochondrial potassium was replaced by tetraethylammonium. Mitochondria were incubated under control conditions or treated with 250 μM Gap19, a peptide that specifically inhibits Cx43-based hemichannels at plasma membranes. Subsequently, 140 mM KCl was added and the slope of the increase in PBFI fluorescence over time was calculated. The slope of the PBFI fluorescence of the control mitochondria was set to 100%. In the presence of Gap19, the mitochondrial potassium influx was reduced from 100 ± 11.6% in control mitochondria to 65.5 ± 10.7% (*n* = 6, *p* < 0.05). In addition to the pharmacological inhibition of Cx43, potassium influx was studied in mitochondria isolated from conditional Cx43 knockout mice. Here, the ablation of Cx43 was achieved by the injection of 4-hydroxytamoxifen (4-OHT; Cx43^Cre-ER(T)/fl^ + 4-OHT). The mitochondria of the Cx43^Cre-ER(T)/fl^ + 4-OHT mice contained 3 ± 1% Cx43 (*n* = 6) of that in control mitochondria (100 ± 11%, *n* = 8, *p* < 0.05). The ablation of Cx43 (*n* = 5) reduced the velocity of the potassium influx from 100 ± 11.2% in control mitochondria (*n* = 9) to 66.6 ± 5.5% (*p* < 0.05). Taken together, our data indicate that both pharmacological inhibition and genetic ablation of Cx43 reduce mitochondrial potassium influx.

## INTRODUCTION

Connexin 43 (Cx43) forms gap junctions between adjacent cardiomyocytes and is thus essential for cell–cell communication. Six Cx43 proteins at the plasma membrane assemble into a hemichannel, and hemichannels open during ischemia and thereby contribute to cell injury (Shintani-Ishida et al., 2007; Clarke et al., 2009). Additionally, Cx43 is present at the inner membrane of cardiomyocyte mitochondria, and cross-linking studies suggest the presence of Cx43-hemichannels within cardiomyocyte mitochondria (Miro-Casas et al., 2009). However, not all cardiomyocyte mitochondria contain Cx43. In contrast to subsarcolemmal mitochondria (SSM), interfibrillar mitochondria (IFM) lack Cx43 (Boengler et al., 2009). An analysis of the impact of Cx43 on mitochondrial function revealed reduced oxygen consumption in mitochondria in which Cx43 was either inhibited by 18α-glycyrrhetinic acid (18αGA) or Cx43-mimetic peptides or in which Cx43 was deleted by conditional knockout (Boengler et al., 2012).

Since mitochondrial potassium fluxes are important for the cardioprotection by ischemic pre- and postconditioning (O’Rourke, 2004; Boengler et al., 2011) and ischemic preconditioning depends on Cx43 (Schwanke et al., 2002), the impact of Cx43 on mitochondrial potassium uptake was studied. In permeabilized wild-type mouse cardiomyocytes, mitochondrial potassium influx was reduced by 18αGA. Also, the replacement of Cx43 by Cx32 – a connexin which forms channels with lower potassium conductance (Harris, 2002) – decreased the potassium influx into permeabilized murine cardiomyocytes (Miro-Casas et al., 2009). In astrocytes, which also contain mitochondrial Cx43, the administration of the Cx43 inhibitors carbenoxolone and 18αGA reduced mitochondrial potassium uptake (Kozoriz et al., 2010).

In the present study, we investigated the importance of Cx43 for potassium uptake in isolated cardiac mitochondria rather than in intact cardiomyocytes. We studied potassium influx in isolated wild-type mouse mitochondria that were treated with Gap19, a novel peptide which specifically targets Cx43-based hemichannels. In addition, mitochondria isolated from conditional Cx43 knockout mice were investigated.

## MATERIALS AND METHODS

### ANIMALS

The present study was performed with approval by the Bioethical Committee of the State of Nordrhein-Westfalen, Germany. It conforms to the *Guide for the Care and Use of Laboratory Animals* published by the US National Institutes of Health (NIH publication No. 85-23, revised 1996).

A dose of 3 mg 4-hydroxytamoxifen (4-OHT) was injected daily for five consecutive days in Cx43^Cre-ER(T)/fl^ mice in which one Cx43 allele had been replaced by the tamoxifen-inducible Cre recombinase. The mice were sacrificed on day 11 after the first injection and mitochondria were isolated from the left ventricles. 4-OHT-treated Cx43^fl/fl^ mice served as a control for potassium measurements, and untreated Cx43^fl/fl^ mice were used as a control for Western blot analysis.

Experiments on the effects of Gap19 on potassium uptake were performed in SSM and IFM from C57/Bl6 mice.

### ISOLATION OF MITOCHONDRIA

Subsarcolemmal mitochondria were isolated as previously described (Boengler et al., 2005). In brief, ventricles were minced in isolation buffer [in mM: sucrose 250; 4-(2-hydroxyethyl)-1-piperazineethanesulfonic acid (HEPES) 10; ethylene glycol tetraacetic acid (EGTA) 1; 0.5% bovine serum albumin (BSA); pH 7.4], homogenized with an Ultra Turrax, and centrifuged at 700 *g* for 10 min. The resulting supernatant was centrifuged at 10,780 *g* for 10 min, and the mitochondrial sediment was re-suspended in isolation buffer without BSA and centrifuged at 7,650 *g* for 10 min. The protein concentration of the isolated mitochondria was determined using the Dc protein assay (Bio-Rad, Hercules, CA, USA) with BSA as standard. For Western blot analysis, the mitochondria were further purified by Percoll gradient ultracentrifugation (30% Percoll in isolation buffer, 34,000 *g*, 30 min).

Subsarcolemmal mitochondria and IFM were isolated as already described (Boengler et al., 2009). Ventricular tissue was washed in buffer A [in mM: KCl 100, 3-(N-morpholino)-propanesulfonic acid (MOPS) 50, MgSO_4_ 5, ATP 1, EGTA 1, pH 7.4] and weighed. Ventricles were minced in 10 ml/g buffer B (buffer A + 0.04% BSA). The homogenate was centrifuged for 10 min at 800 *g* and the resulting supernatant (for SSM isolation) for 10 min at 8,000 *g*. The sediment was re-suspended in buffer A, washed, and re-suspended in a small volume of buffer A. The sediment of the first centrifugation (used for isolation of IFM) was re-suspended in buffer B (10 ml/g tissue). Nagarse (8 U/g) was added and incubated for 1 min on ice. The tissue was homogenized and centrifuged for 10 min at 800 *g*. The supernatant was centrifuged for 10 min at 8,000 *g*. The mitochondria in the sediment were re-suspended, washed in buffer A, and finally re-suspended in a small volume of buffer A.

### MITOCHONDRIAL POTASSIUM INFLUX

Mitochondria were loaded with 10 μM PBFI-AM [acetoxymethyl ester of PBFI (potassium-binding benzofuran isophthalate); Sigma-Aldrich, Heidenheim, Germany] diluted 2:1 with 20% pluronic F127 for 10 min at 25°C according to the protocol by Costa et al. (2006). Three volumes of tetraethylammonium (TEA) buffer (in mM: sucrose 175, TEA-Cl 50, HEPES 10, pyruvate 5, malate 5, succinate 5, P_i_ 5, EGTA 0.1, and MgCl_2_ 0.5 for experiments with conditional Cx43 knockout mitochondria; TEA-Cl 120, HEPES 10, succinate 10, Na_2_HPO_4_ 5, EGTA 0.1, MgCl_2_ 0.5, rotenone 5 μM, oligomycin 0.67 μM, pH 7.2 for experiments with Gap19) were added, and the mitochondria were incubated for 2 min. Subsequently, the mitochondria were washed twice in isolation buffer and the protein concentration was measured using the Dc protein assay (Bio-Rad, Hercules, CA, USA). A sample of 200 μg mitochondrial proteins (SSM and IFM) was incubated for 30 min at 4°C with 250 μM of the Cx43-hemichannel blocking peptide Gap19 or under control conditions. In addition, untreated mitochondria from Cx43^fl/fl^ and Cx43^Cre-ER(T)/fl^ + 4-OHT mice (SSM) were studied. Mitochondria (100 μg/ml) were added to isolation buffer supplemented with 1 μg/ml oligomycin (inhibits the ATP synthase), 50 μM glibenclamide (blocks the mitochondrial ATP-dependent potassium channel; control and conditional Cx43 knockout mice), and 1 μM cyclosporin A (inhibits opening of the mitochondrial permeability transition pore; Gap19 experiments). The uptake of potassium into the mitochondria was induced by adding 140 mM KCl. The PBFI fluorescence was measured in a Cary Eclipse Fluorescence Spectrophotometer (Varian, Mulgrave, Australia) at alternating excitation wavelengths of 340 (maximum potassium sensitivity of the probe) and 380 nm (isosbestic point of the probe), respectively, and an emission wavelength of 500 nm at 25°C. The maximal slope of the PBFI fluorescence after the KCl pulse was determined, and the maximal slope for the control mitochondria was set to 100%.

### WESTERN BLOT ANALYSIS

Mitochondrial proteins were extracted in 1× Cell Lysis Buffer [Cell Signaling, Beverly, MA, USA, containing in mM: Tris 20, NaCl 150, ethylenediaminetetraacetic acid (EDTA) 1, EGTA 1, sodium pyrophosphate 2.5, β-glycerolphosphate 1, Na_3_VO_4_ 1, phenylmethanesulfonyl fluoride (PMSF) 1, 1 μg/ml leupeptin, 1% Triton X-100, pH 7.5, supplemented with complete protease inhibitors (Roche, Basel, Switzerland)]. After centrifugation at 13,000 *g* for 10 min at 4°C the supernatants were collected, and the protein concentrations were determined using the Dc protein assay (Bio-Rad, Hercules, CA, USA). Right ventricular or mitochondrial proteins (20 μg) were electrophoretically separated on 10% SDS-PAGE (sodium dodecyl sulfate-polyacrylamide gel electrophoresis) and transferred to nitrocellulose membranes. After blocking, the membranes were incubated with rabbit polyclonal anti-rat Cx43 (Invitrogen, Carlsbad, CA, USA) or rabbit-polyclonal anti-human manganese superoxide dismutase (MnSOD, Upstate, Lake Placid, NY, USA). After incubation with the respective secondary antibodies, immunoreactive signals were detected by chemiluminescence (SuperSignal West Femto Maximum Sensitivity Substrate, Pierce, Rockford, IL, USA) and quantified with the Scion Image software (Frederick, MD, USA).

### STATISTICS

Data are presented as mean values ± SEM. Western blot data and the velocities of mitochondrial potassium uptake were compared by Student’s *t*-test.

## Results

**FIGURE 1:**
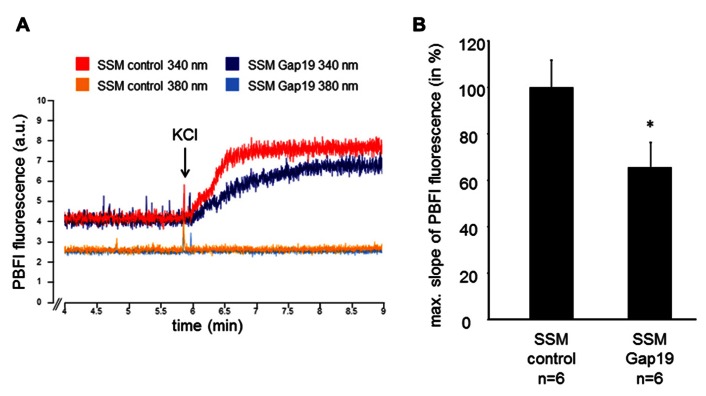
**Mitochondrial potassium uptake is decreased by Gap19**. **(A)** Original traces showing PBFI fluorescence in arbitrary units (a.u.) before and after addition of 140 mM KCl of control- or Gap19-treated SSM at 340 and 380 nm excitation and 500 nm emission, respectively. **(B)** Bar graphs represent the maximal slope of the PBFI fluorescence at 340 nm excitation and 500 nm emission of control and Gap19-treated mitochondria. *p < 0.05.

The velocity of the mitochondrial potassium influx was measured in wild-type SSM under control conditions and after incubation with 250 μM Gap19, a peptide that specifically inhibits Cx43-based hemichannels at plasma membranes, at excitation wavelengths of 340 and 380 nm, respectively (**Figure 1**). In the presence of Gap19, the velocity of the mitochondrial potassium influx (340 nm) was reduced from 100 ± 11.6% in control mitochondria to 65.5 ± 10.7% (*n* = 6, *p* < 0.05). At 380 nm excitation, which represents the isosbestic point, the addition of KCl did not affect the PBFI fluorescence (6.5 ± 0.8% control vs. 7.8% ± 1.0% Gap19 treatment, *n* = 6, *p* = ns). In IFM, which do not contain Cx43, Gap19 treatment had no influence on the velocity of the mitochondrial potassium uptake (100 ± 11.8% IFM control vs. 106.2 ± 22.2% IFM Gap19-treated, *n* = 5, *p* = ns).

**FIGURE 2:**
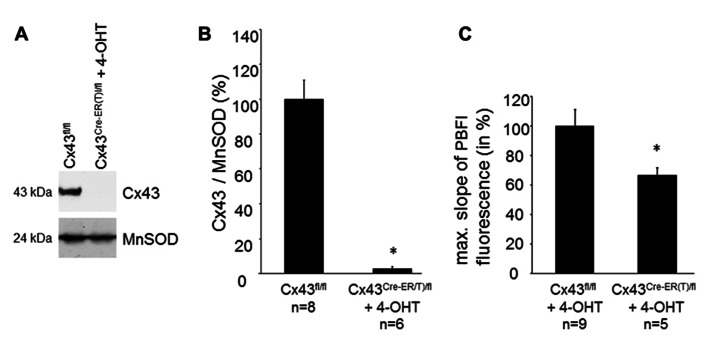
**Genetic ablation of Cx43 reduces mitochondrial potassium uptake**. **(A)** Western blot analysis was performed for Cx43 and the mitochondrial marker protein MnSOD on proteins isolated from SSM of Cx43^fl/fl^ and Cx43^Cre-ER(T)fl^ + 4-OHT mice. **(B)** Bar graphs represent the mitochondrial Cx43 content normalized to MnSOD in Cx43^fl/fl^ control mice, which were set to 100%, and 4-OHT-treated Cx43^Cre-ER(T)/fl^ mice. *p < 0.05. **(C)** Bar graphs represent the maximal slope of the PBFI fluorescence at 340 nm excitation and 500 nm emission of 4-OHT-treated Cx43^fl/fl^ and Cx43^Cre-ER(T)/fl^ mitochondria. *p < 0.05.

The Cx43 content in SSM was determined in Cx43^Cre-ER(T)fl^ mice treated with 4-OHT and in Cx43^fl/fl^ control mice (**Figures 2A,B**). Western blot analysis demonstrated a reduction in mitochondrial Cx43 from 100 ± 10.7% in Cx43^fl/fl^ mice (*n* = 8) to 3.2 ± 1.1% in Cx43^Cre-ER(T)/fl^ + 4-OHT mice (*n* = 6, *p* < 0.05). To exclude effects of 4-OHT treatment on mitochondrial function, potassium influx was measured in mitochondria isolated from 4-OHT-treated Cx43^fl/fl^ and Cx43^Cre-ER(T)/fl^ mice. The reduction of the mitochondrial Cx43 content was associated with a reduction in the maximal slope of the PBFI fluorescence from 100 ± 11.2% in Cx43^fl/fl^ + 4-OHT mitochondria (*n* = 9) to 66.6 ± 5.5% in Cx43^Cre-ER(T)/fl^ + 4-OHT mitochondria (*n* = 5, *p* < 0.05, **Figure 2C**).

## DISCUSSION

The present study addressed the role of Cx43 in mitochondrial potassium uptake and demonstrated that both inhibition and genetic ablation of mitochondrial Cx43 reduce the velocity of the mitochondrial potassium influx specifically in SSM.

The electrochemical gradient drives an inward potassium flux into mitochondria. For cardiac mitochondria, the ATP-dependent potassium (mitoK_ATP_) channel is of major importance. This channel is activated by pharmacological agents such as diazoxide, whereas glibenclamide and ATP inhibit mitoK_ATP_ channel activity. The molecular identity of the mitoK_ATP_ channel has not yet been definitely established. However, data suggest mitochondrial localization of sarcolemmal potassium channel subunits (Ye et al., 2009), and, most recently, the contribution of the ROMK (renal outer medullary potassium channel) to the mitoK_ATP_ channel (Foster et al., 2012).

Opening of the mitoK_ATP_ channel is important for the cardioprotection afforded by ischemic preconditioning (O’Rourke, 2004; Ardehali and O’Rourke, 2005), presumably via the formation of low amounts of reactive oxygen species that function as signaling molecules (Pain et al., 2000). Cx43 is involved in the cardioprotection conferred by mitoK_ATP_ channel opening, since reactive oxygen species formation and subsequent infarct size reduction by diazoxide are lost in Cx43-deficient mice (Heinzel et al., 2005).

The present study addressed the contribution of mitochondrial Cx43 to potassium influx. Experiments were performed in the presence of glibenclamide in order to allow the analysis of putative Cx43-based channels and separate them from effects of Cx43 on mitoK_ATP_ channels.

Previous data showed that in permeabilized mouse cardiomyocytes Cx43 contributes to mitochondrial potassium uptake (Miro-Casas et al., 2009). In this study, the Cx43 inhibitor 18αGA was employed, and cardiomyocytes were used in which Cx43 had been replaced by Cx32. The aim of the present study was to investigate the importance of Cx43 for mitochondrial potassium refilling in more detail. Therefore, isolated mitochondria from control and conditional Cx43 knockout mice were used. The ablation of Cx43 was achieved by the administration of 4-OHT, which may impact on mitochondrial function, especially on mitochondrial calcium homeostasis (Lobaton et al., 2005). However, previous data demonstrated that the 4-OHT treatment used here has no influence on mitochondrial oxygen consumption (Boengler et al., 2012). To exclude putative effects of 4-OHT on mitochondrial potassium uptake, data obtained in mitochondria from conditional Cx43 knockout mice were compared to those obtained from 4-OHT-treated control mice. Our data demonstrate that mitochondria that contain only minimal amounts of Cx43 (about 3% of the amount of control mitochondria) have a reduced velocity of potassium refilling. Therefore, our data confirm the importance of Cx43 for mitochondrial potassium uptake.

In addition to the chronic scenario of the Cx43 knockout mice, the acute situation was analyzed in which mitochondrial Cx43 was inhibited by Gap19. Gap19 is a peptide derived from nine amino acids of the Cx43 cytoplasmic loop. This peptide inhibits single channel Cx43-hemichannel currents at plasma membranes but has no effect on gap junction channels or Cx40/pannexin-1-dependent hemichannels as determined by calcium-induced ATP release. Gap19 binds to the carboxy terminus of Cx43 and thereby prevents interactions between the carboxy terminus and the cytoplasmic loop (Wang et al., 2013). In isolated cardiomyocytes, Gap19 reduced cell swelling and cell death following simulated ischemia/reperfusion and reduced infarct size in mouse hearts *in vivo* (Wang et al., 2013). The specificity of Gap19 for Cx43-based hemichannels makes this peptide an excellent tool to study the role of Cx43 in mitochondrial potassium uptake. Since the carboxy terminus of mitochondrial Cx43 is directed toward the intermembrane space (Boengler et al., 2009), Gap19 does not have to enter the mitochondrial matrix in order to interact with Cx43.

In SSM isolated from wild-type mice, Gap19 induced a decrease in the velocity of mitochondrial potassium influx. In IFM, which do not contain Cx43 (Boengler et al., 2009), Gap19 had no effect, demonstrating the specificity of the peptide for Cx43.

The present experiments were performed in the presence of glibenclamide, which inhibits potassium fluxes through mitoK_ATP_ channels (Inoue et al., 1991; Paucek et al., 1992); therefore, it is unlikely that the delayed potassium influx in Cx43-deficient and Gap19-treated mitochondria is due to effects of Cx43 on these channels. However, several other channels also contribute to the mitochondrial potassium cycle, among them calcium-dependent potassium channels, the mitochondrial Kv1.3 potassium channel, and the two-pore potassium channel TASK-3 (for review, see Szabo et al., 2012). Since in the present study only total mitochondrial potassium fluxes were measured, it was not possible to distinguish whether mitochondrial potassium influx occurs through putative Cx43-based hemichannels or through an indirect modulation of the above mentioned potassium channels. Whether or not mitochondrial Cx43 forms a channel similar to those at the plasma membrane remains to be established. However, cross-linking studies of mitochondrial proteins with subsequent Western blot analysis for Cx43 revealed immunoreactive signals at a molecular weight typical for Cx43-based hexamers (Miro-Casas et al., 2009).

Taken together, both the genetic ablation of Cx43 in conditional knockout mice and the acute inhibition of mitochondrial Cx43 by Gap19 demonstrate reduced velocities of mitochondrial potassium uptake. These findings substantiate the impact of Cx43 on mitochondrial potassium fluxes.

## Conflict of Interest Statement

The authors declare that the research was conducted in the absence of any commercial or financial relationships that could be construed as a potential conflict of interest.
